# Comparison of physical activity and quality of life in home haemodialysis (HHD) patients versus conventional in-centre haemodialysis (ICHD) patients: the observational, longitudinal, prospective, international, multicentric SeCoIA study protocol

**DOI:** 10.1186/s12882-020-02127-7

**Published:** 2020-11-23

**Authors:** Natalia Target, Cécile Courivaud, Pierre Antoine Michel, Salima Daoud, Michel Thomas

**Affiliations:** 1 Department of Nephrology and Dialysis, Centre hospitalier- Site La Roche/Yon, La Roche-sur-Yon, France; 2 Department of Nephrology, Dialysis and Renal Transplantation, University of Franche-Comté, Besançon, France; 3 Department of Nephrology and Dialysis, Assistance Publique Hôpitaux de Paris, Hôpital Tenon, Paris, France; 4 Monitoring Force Group, Maisons-Laffitte, France; 5 Physidia SAS, Saint-Barthélémy-d’Anjou, France

**Keywords:** End-stage renal disease, Home haemodialysis, Physical activity, 3-axis accelerometer, Quality of life, Restless leg syndrome

## Abstract

**Background:**

Home haemodialysis (HHD), has shown improved clinical outcomes, as well as a better quality of life, compared to conventional in-centre haemodialysis (ICHD) but still has a global low prevalence among end-stage renal disease patients. Haemodialysis (HD) patients tend to be sedentary but only few studies, mainly in North American ICHD patients, have evaluated the level of activity in HD patients.

**Methods:**

SeCoIA is an observational, longitudinal, prospective, international, multicentric, study, conducted in metropolitan France and Belgium. The main objective of the study is to quantify the physical activity measured by the total daily number of steps, in HHD patients compared to ICHD patients. The SeCoIA study will include 80 HHD patients and 80 ICHD patients,. Secondary objectives will be to characterize the HHD population and to confirm HHD efficiency on clinical parameters, as well as quality of life (QoL), in current practice. Physical activity will be measured by a 3-axis accelerometer. Accelerometers have been shown to provide accurate information, on both physical activity and sedentary behaviour. Patients will be instructed to wear the device and complete a patient diary 7 consecutive days after inclusion and the first week of each month for 12 months. Decision to undergo HDD or ICHD is independent of the study and follow-up frequency remains at the discretion of the physician/centre. QoL and quality of sleep will be respectively assessed by the Kidney Disease Quality of Life 1.2 (KDQOL™) and the Pittsburg Sleep Quality index (PSQI) questionnaires at inclusion, 6- and 12-month visits. Patients presenting a restless leg syndrome (RLS) will also complete the International Restless Legs Syndrome rating scale (IRLS) questionnaire.

**Discussion:**

The SeCoIA study will be the first large cohort study (160 patients) evaluating physical activity, objectively measured with a 3-axis accelerometer, in HHD versus ICHD patients. The present study will also include a comparison of QoL with a focus on RLS between HHD and ICHD. It is anticipated that HHD patients will have an improved physical activity and QoL which should encourage physicians to further promote HHD.

**Trial registration:**

Clinical trial NCT03737578 study registered on November 9, 2018 (Retrospectively registered).

## Background

End-stage renal disease (ESRD) is a major concern of global public health. The Global Burden of Disease 2015 study estimated that, worldwide, in 2015, 1.2 million people died from ESRD, an increase of 32% since 2005 [1]. ESRD is a serious complication of many pathologies, such as diabetes, hypertension and the number of patients treated for ESRD keeps increasing [2]. ESRD is an important economic burden and is associated with a decrease in quality of life [2].

In France, according to the REIN report (French Network of Epidemiology and Information on Kidney Diseases), on December 31, 2016, 84,683 patients were receiving a renal replacement therapy, 46,872 (55%) were on dialysis and 37,811 (45%) were living with a functional renal transplant. Among dialysis patients, 93.7% received haemodialysis (HD) of which 54.6% in centres (ICHD) and only 0.8% at home (HHD) [3].

Even though several studies have shown an improved efficiency of dialysis, as well as a better quality of life in these patients, HHD prevalence remains low [4, 5]. FHN (Frequent Haemodialysis Network) [6] and FREEDOM (Following Rehabilitation, Economics and Everyday-Dialysis Outcome Measurements) [7] are the main studies that compared the effects of short-daily (ICDH and HHD) to conventional thrice-weekly ICHD, focusing on clinical and economic benefits. Both studies were conducted in the United States. In the FHN trial, frequent (6-times per week) ICHD, as compared to conventional (3-times per week) ICHD, was associated with significant benefits, in term of mortality and left ventricular mass [6]. FREEDOM, a prospective multicentre observational study investigating the benefits of daily (6-times per week) HHD, reports improved clinical parameters, as well as psychological state, in HHD versus ICHD patients. Among the clinical parameters, patients showed a decrease in restless legs syndrome (RLS), as well as a decrease in post-dialysis recovery time [8, 9].

Few data are available on physical activity, in HD patients and the rare studies are mostly small and performed in ICHD [10–14]. One study with 43 subjects (23 kidney transplant recipients and 20 ICHD patients) in Brazil, measuring physical activity with a multiaxial accelerometer, showed that ICHD patients tend to be more sedentary than kidney transplant recipient [13]. On the other hand, in the UK, one study with only 28 HHD patients showed no difference in physical activity, evaluated by 2 questionnaires, compared to healthy controls [12]. In the EXCITE (EXerCise Introduction To Enhance Performance in Dialysis) trial, a multicentric randomized controlled trial in Italian centres, poor physical performance predicted a high risk of mortality, cardiovascular events and hospitalizations in this population of 296 dialysis patients [15]. Furthermore, in a secondary analysis of EXCITE, a home-based exercise program showed improvement of physical performance of those patients [16].

To our knowledge, no study focuses on the physical activity of HHD compared to ICHD patients. SeCoIA (Self COnvective haemodialysis Investigation Analysis), is an, observational, longitudinal, prospective international, multicentric study, that will compare physical activity, measured with a connected 3-axis accelerometer, between HHD and ICHD. It has to be noted that the use of an accelerometer classifies the study as interventional in France. The other objectives of the study are to characterize HHD patients and to confirm, in current practice in France and in Belgium, the efficiency of HHD on clinical parameters, as well as quality of life.

## Methods

### Study design

**Fig. 1:**
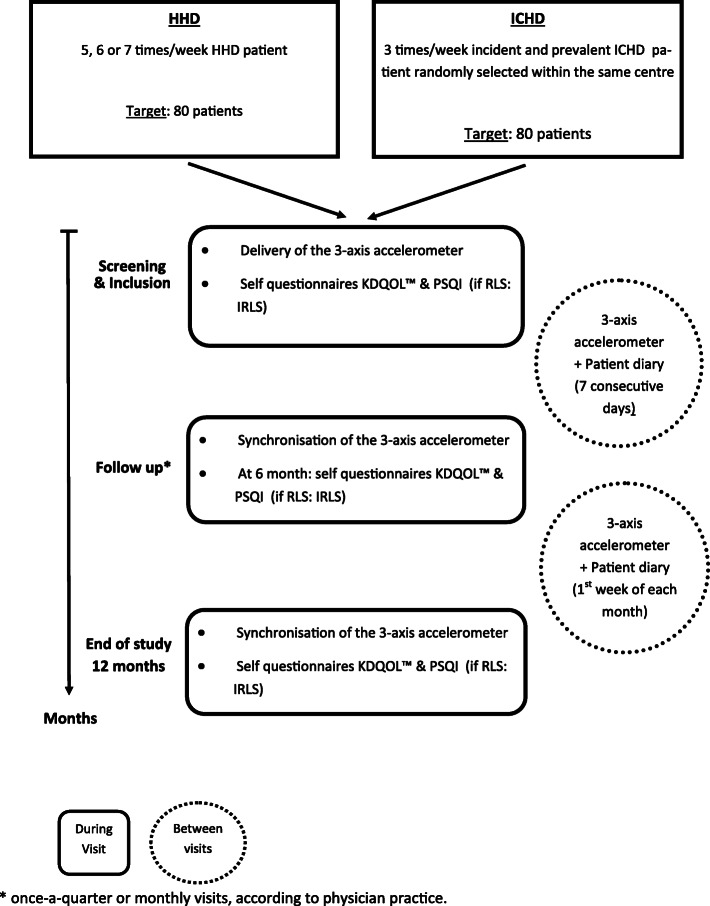
Flow chart of the SeCoIA study

SeCoIA is an observational, longitudinal, prospective, international, multicentric, cohort study comparing HHD patients to ICHD patients, conducted with metropolitan French and Belgian HD centres (Fig. 1).

Eligible HHD patients are adult patients, starting an HHD training or about to start an HHD. ICHD patients will be randomly selected (ratio 1:1) in each center among patients aged less than 80 years.

Decision to undergo HHD or ICHD is independent of the study. All included patients will be followed-up for 12 months or kidney graft or withdrawal of informed consent, which event occurred first.

### Objectives

The main objective of the SeCoIA study is to evaluate the physical activity in the daily life of patients in HHD (5 to 7 times a week) in comparison with 3 times a week ICHD patients.

Secondary objectives include: 1) comparison of physical activity in HHD patients on the days with HD or without HD; 2) description of the prescription procedures of the HHD; 3) comparison of blood pressure changes between the two HD modalities; 4) comparison of the frequency, duration and reasons of hospitalizations between the two HD modalities; 5) comparison of cardiovascular risk between the two HD modalities; 6) comparison of the quality of life of the patients between the two HD modalities; 7) evaluation of the quality of the sleep of the patients between the two HD modalities; 8) comparison of the percentage of patients with RLS and the severity of RLS between the two HD modalities; 9) comparison of the evolution of drug intake (iron, erythropoietin stimulating agents, phosphate-binding agents, vitamin D, calcium supplementation, calcimimetics, calcium carbonate, bicarbonates, potassium binders, oral anticoagulants, heparin, antihypertensives, lipid-lowering drugs, glucose lowering drugs, analgesics, drugs related to RLS and sleep disorders) between the two HD modalities; 10) evaluation of the number of subjects who stopped the HHD technique and causes of discontinuation; 11) comparison of characteristics (age, sex, body mass index and blood pressure) in patients wearing the 3-axis accelerometer or not.

### Study population

SeCoIA will include 160 male and female adult patients, 80 HHD patients and 80 ICHD patients.

#### Inclusion criteria

1. Male or female aged 18 years or over,
2. Patient who dated and signed the consent form,
3. Affiliation to Social Security System,

**Table:** 

HHD patient	ICHD patient
Patient who begins or will begin HDD training (5, 6 or 7 times per week).	ICHD (3 times per week); incident or prevalent patient randomly selected within the same centre.

To avoid a great imbalance between the 2 groups, random selection of ICHD subjects will be restricted to patients aged less than 80 years old at the time of sampling.

#### Non-inclusion criteria

1. Mobility impairment (use of wheelchair, crutch, walker, cane ...),
2. Active neoplasia,
3. Predicted life expectancy of less than one year,
4. Significant reading or writing impairment,
5. Participation to another clinical trial or other interventional study,
6. History of mental instability, major cognitive impairment within the last 5 years, or major psychiatric condition not adequately controlled or stable under pharmacological treatment.

Patients who refuses to wear the 3-axis accelerometer or receiving new HD after kidney graft failure can be included in the SeCoIA study.

### Study procedures

The investigator completes a paper case report form (CRF) at inclusion and at least once a quarter or during a monthly visit. The frequency depends on physician current practice.

At the end of the inclusion visit (V0), participants (except in case of explicit refusal) will receive a connected 3-axis accelerometer (FITBIT®Flex2™); 3-axis accelerometers have been shown to be accurate with a very low variability compared to other instruments [17] and can provide large amount of data on light-intensity physical activity and sedentary behaviour [18]. The patients will be instructed to wear the device on the arm opposite to arterio-venous fistula for 7-consecutive days (at least from awakening to bedtime), after inclusion and the first week of each month, and to complete a patient diary including start and end dates of 3-axis accelerometer use, days of HD and start and end body weight on the first day of HD of the same week. The device will be synchronized during all follow-up visits (V1-V12). Collected data will include the total number of steps per day, sedentary behaviour (minutes of light, moderate or high activity), sleep pattern (start and end time of sleep, minutes of sleep, number of waking-up).

The patient diary includes also questions on physical activity at inclusion and at the end of the study (adapted from [19]).

Quality of life (KDQOL 1.2 Kidney Disease Quality of Life) and quality of sleep PSQI (Pittsburg Sleep Quality index) questionnaires are to be filled, during a visit, by the patient, upon inclusion and then at 6 months and at 12 months or in case of premature withdrawal. For patients with a diagnosed restless leg syndrome (RLS) according to the investigator, the International RLS (IRLS) questionnaire will be filled at inclusion, 6 and 12 months or in case of premature withdrawal. If participant develop a RLS during the course of the study, the IRLS will also be completed at the visit with first report of RLS, and then 6 months and 12 months after inclusion.

When a Physidia device is used, some modalities of HD (duration of dialysis, blood pressure) may be collected from Physidia SAS.

### Study assessments

#### Primary outcome

The main criterion is the average number of total steps per day at 3, 6, 9 and 12 months.

#### Secondary outcome measures

1. Average number of steps per day among ICHD patients on dialysis days and on non-dialysis days;
  Characteristics of HHD (Table 1);

**Table 1:** Study schedule

collected Data	Inclusion	Monthly visit^a^	Quaterly VISIT	End of Study 12 Months^b^
Selection Criteria^c^	x			
Demography^d^	x			
Social Characteristics^e^	x	x	x	x
Medical History	x			
Drugs^f^	x	x	x	x
Blood pressure^g^	x	x	x	x
Weight pre and post dialysis	x	x	x	x
Dialysis characteristics^h^	x		x	x
Laboratory^i^	x	x	x	x
Synchronization Accelerometer		x	x	x
Hospitalizations/ Complications		x	x	x
Adverse Events		x	x	x
KDQOL, PSQI	x		6-month visit only	x
IRLS (if applicable)	x		6-month visit only	x
End of Study Form^b^				x

x: Collected data

^a^ According to centre/physician standard practice

^b^ or in case of early termination

^c^ Written consent, eligibility criteria, date of inclusion visit

^d^ Age, sex, height, smoking status

^e^ Professional activity, commute modalities to come to the centre and home- centre distance

^f^ Iron, erythropoietin stimulating agents, phosphate-binding agents, vitamin D, calcium supplementation, calcimimetics, calcium carbonate, bicarbonates, potassium binders, oral anticoagulants, heparin, antihypertensives, lipid-lowering drugs, glucose lowering drugs, analgesics, drugs related to RLS (ropinirole, pramipexole, rotigotine) and sleep disorders (anxiolytics, muscle relaxants, hypnotics)

^g^ Blood pressure before / after dialysis (collection of the last 3 available measurements at 3 different days)

^h^ For ICHD patient: Dialysis method (HD, pre-dilution HDF, post-dilution HDF), total convective volume per session for HDF patients, plasma volume control technique (yes / no); for HDD patients: Training start date, convective volume prescribed per session, dialysate flow

^i^ Blood: hemoglobin, glycated hemoglobin, calcemia, phosphoremia, PTH, bicarbonates, alkaline phosphatase, ferritin, transferrin saturation, albumin, natremia, predialytic β2microglobulin; urinary assessment with the method of collection (sample, 12 h or 24 h urine): creatinuria, urea, proteinuria, microalbuminuria
2. Evolution of the blood pressure during the study period (V0-V12);
3. Frequency and duration of hospitalizations during the study period (V0-V12);
4. Cardiovascular complications;
5. Quality of life of the patients estimated with questionnaire KDQOL-SF version 1.2 at inclusion, 6 and 12 months. KDQOL 1.2 questionnaire has been validated and is commonly used as a measure of quality of life in dialysis patients [20];
6. Quality of the sleep of the patients through PSQI version 1.0 questionnaire evaluated at inclusion, 6 and 12 months. PSQI questionnaire assesses sleep quality over a one-month period [21];
7. Drug intake during the study period (posology of drugs linked with ESRD);
8. Percentage of premature withdrawal from the study and reasons;
9. Percentage of patients with RLS during the study period and assessment of severity with an IRLS (International Restless Legs Syndrome rating scale) questionnaire [22].

### Study organisation

The scientific committee (CC and PAM) of the SeCoIA study validated the scientific rational, the objectives, the methodology and will review and validate the statistical analysis plan and the statistical report. All publications of the results will have to be approved by the scientific committee and the coordinator of the study (NT). Monitoring is performed by on-site visits, as well as remote monitoring by trained Clinical Research Associates, following ICH-GCP guidelines and according to a monitoring plan. Review of data collected in the CRF and direct access to patient medical information will ensure data verification. Patient confidentiality will be ensured under the French MR-001 reference methodology [23] and data confidentiality under the EU General Data Protection Regulation [24].

The SeCoIA protocol does not influence medical practice, drug prescription and dialysis modalities remain at the entire discretion of the participating investigators. However, all adverse events (AE) and serious adverse events (SAE) that may be related to a drug or the medical device (e.g. dialysis device) must be reported to the local authorities in a timely manner, as per French and Belgian regulation. All AEs will be recorded in the CRF.

Monitoring, data management and statistical analysis will be performed by Monitoring Force Group, a Contract Research Organisation (CRO).

### Statistical methods

#### Sample size

We hypothesize a difference in the average number of steps of 1000 steps per day between HHD and ICHD patients with a standard deviation of 2178 steps [11, 13] identical in the 2 groups. With 5 repeated measures, a first autoregressive covariance variance matrix structure, an α risk of 5% and a power of 90%, the number of subjects depends on the correlation of the number of steps between 2 records for a given subject. No published data are available, also the sample size was calculated using different estimates of correlation from 0.4 to 0.6. Using a 0.6 correlation, 53 patients in each group are necessary. To take into account the number of non-evaluable subjects and to be able to adjust on confounding factors, the final sample is estimated to be 80 subjects in each group.

#### Statistical analysis

Standard descriptive statistics will be used to summarize the data in HHD and in ICHD patients. All results will be blinded to the study site. All statistical tests will be two-sided at the 0.05 significant level. No replacement of missing data will be done except for the main criterion of analysis where a missing quarterly value will be replaced by the last value available for that quarter [Last Observed Carried Forward (LOCF)]. If no data is available for a given quarter, no missing value will be replaced for that quarter.

The average number of steps per day at each recording will be used for the analysis of the main criterion. A repeated measurement analysis will be performed using generalized linear mixed model with adjustment on confounding factors at inclusion (age, sex, duration since start of HD, body mass index, diabetes mellitus, arterial hypertension and heart failure) as fixed effects and the intercept and the slope of time of recording as random effects. The covariance structure with the minimum Akaike information criterion (AIC) will be selected. Two-way interactions will be tested.

### Ancillary studies

A medico-economic and ancillary study may be conducted using data collected in this study (hospitalizations, cardiovascular complications, dialysis complication, surgeries, drug intake, medical commute, …) to assess costs and with possibly additional data collection. A separated protocol will be developed for this ancillary study, if any.

## Discussion

Dialysis patients tend to be more sedentary than healthy subjects or transplant recipients, despite all the potential beneficial effects of exercise that this population could benefit from [13, 25, 26]. In a recent survey, using self-reporting questionnaires in 2 Canadian centres, the comparison of physical activity depending on dialysis modality, showed that HHD patients had a possible trend of higher moderate physical activity than ICHD patients [27]. However, only 26 HDD patients were included where the SeCoIA study will include 80 HHD patients. Also, physical activity will be measured by a 3-axis accelerometer to ensure accurate quantification. Thus, the design of the SeCoIA study will allow to have an evaluation of physical activity in a large cohort of HHD patients (80 patients), over a long period of time (12 months). However, HHD tends to be moderately prescribed in older patients and ICHD patients are often elderly patients [28]. Nonetheless, a small study in New Zealander patients over 65 years, showed that even older adults could benefit from the at-home dialysis modalities in term of quality of life [29]. Therefore, it was initially planned to match ICHD and HHD patients in each centre on age, sex and HD duration. However, the matching was so difficult that it was decided to draw a random sample of ICHD patients restricted to patients younger than 80 years. Even if the bias cannot be fully eliminated, in the SeCoIA study, the statistical analysis will be adjusted on main confounding factors including age.

In the SeCoIA study, choice of HD modality is independent of the study and decided before inclusion in the study. The ancillary study may help change the prescription pattern in France if results confirm clinical and economic benefits, as previously demonstrated in other countries [7, 8, 30].

Daily HHD is associated with an increase in dialysis efficiency, as well as a better quality of life [4, 5], but still has a very low prevalence worldwide (2% in USA [31], 0.3% in Canada [32], 0.7% in France [3] 1–2% in the UK [33]), with the exception of Australia and New Zealand with a prevalence of respectively 9.6 and 22.8% [30, 34]. Large observational cohort studies generally report, at the clinical level, lower mortality and decreased hypertension and at the medico-economic level, decreased hospitalization duration [4]. A recent American study on more than 5000 chronic dialysis patients, analysed the quality of life depending on dialysis modality [5]. At baseline, home modality (HHD and peritoneal dialysis) patients had higher KDQOL scores than ICHD. Over time, patients who did not change dialysis modalities tended to maintain the same quality of life level. Furthermore, it was shown that patients switching from at-home to in-centre had a significant decrease of physical functioning over time but those patients may have been sicker with higher comorbidities [5]. The present study will allow to compare QoL between HHD and ICHD at inclusion and its evolution over a 12-month period.

RLS remains a debilitating burden for patients and is relatively common in HD population with a prevalence of 6–62% with a poor quality of sleep and a decrease in quality of life [7]. RLS has been associated with increased cardiovascular diseases [35] but not with increased mortality [36]. In the FREEDOM study, an intermediate report showed that over a 12-month period, short daily HD was associated with an improvement of RLS and quality of sleep [9]. A randomized controlled trial on subjects who presented a RLS showed that a conditioning program of aerobic and lower-body resistance 3-days per week for 12 weeks, improved the severity of symptoms [37]. The authors did not specify if HD subjects were included in their study but the HD population especially the HHD patients, could benefit from an exercise program. However, very little information is available, especially on the French HHD population [38]. In our study, one of the secondary outcomes of the SeCoIA trial is to estimate the frequency of RLS, to assess the severity of the symptoms and eventually to establish a link between RLS and physical activity, in the HHD population.

In conclusion, the SeCoIA study will be the first large cohort study (80 HDD patients versus 80 ICHD patients) on the level of physical activity, objectively measured with a 3-axis accelerometer, in HD patients (HHD patients versus ICHD patients). Physical activity will be analysed with regards to the quality of life in those patients with a focus on RLS. It is anticipated that physical activity will be higher, and that QoL will remain higher in HHD patients compared to ICHD patients. This improvement should encourage physician to further promote HHD.

## Data Availability

Data sharing is not applicable to this article as no datasets have been generated or analysed from the current study yet.

## Abbreviations

(S)AE: (Serious) Adverse events
CRF: Case report form
ESRD: End-stage renal disease
EXCITE: EXerCise Introduction To Enhance Performance in Dialysis
FHN: Frequent Haemodialysis Network
FREEDOM: Following Rehabilitation, Economics and Everyday-Dialysis Outcome Measurements
HD: Haemodialysis
HHD: At-home haemodialysis
ICHD: In-centre haemodialysis
IRLS: International Restless Legs Syndrome rating scale
KDQOL: Kidney Disease Quality of Life Instrument
PSQI: Pittsburg Sleep Quality index
SeCoIA: Self COnvective haemodialysis Investigation Analysis
RLS: Restless Legs Syndrome
